# Association of the prognostic nutritional index with in-hospital adverse cardiovascular events among emergency department patients with acute heart failure

**DOI:** 10.3389/fnut.2026.1949478

**Published:** 2026-10-20

**Authors:** Xiao-Peng Zhang, Wei-Wei Guo, Bin Li

**Affiliations:** Department of Emergency Medicine, Xinxiang Central Hospital, The Fourth Clinical College of Henan Medical University, Xinxiang, Henan, China

**Keywords:** acute heart failure (AHF), emergency department, in-hospital adverse cardiovascular events, nutritional status, prognostic nutritional index (PNI), risk prediction

## Abstract

**Background:**

Acute heart failure (AHF) is prone to early clinical deterioration. The prognostic nutritional index (PNI), derived from serum albumin and lymphocyte count, is readily available. We evaluated the association of early PNI with incident in-hospital adverse cardiovascular events and its incremental prognostic value.

**Methods:**

This retrospective cohort included consecutive adults presenting to the emergency department with AHF from March 2022 through March 2026. PNI was calculated from measurements obtained within 24 h and classified as low (<45) or high (≥45). The primary outcome was a post-index composite of all-cause death, cardiogenic shock, sustained ventricular arrhythmia, cardiac arrest, acute myocardial infarction, or worsening AHF requiring treatment escalation. Multivariable logistic regression evaluated associations; missing covariates were handled using multiple imputation. Discrimination was quantified by the area under the receiver operating characteristic curve (AUC).

**Results:**

Of 367 patients (156 low PNI; 211 high PNI), 112 (30.5%) experienced the primary outcome. Events occurred in 43.6% and 20.9%, respectively (*P* < 0.001). After adjustment, low PNI remained associated with higher odds of the primary outcome (odds ratio [OR], 1.92; 95% confidence interval [CI], 1.10–3.35; *P* = 0.022). Each 5-point PNI increase was associated with lower odds (OR, 0.70; 95% CI, 0.56–0.90; *P* = 0.003). PNI alone yielded an AUC of 0.714. Adding PNI increased the conventional model AUC from 0.776 to 0.829 (increase, 0.053; 95% CI, 0.021–0.085; *P* = 0.001); bootstrap-corrected AUCs were 0.752 and 0.803, respectively.

**Conclusions:**

Low PNI was associated with higher adjusted odds of in-hospital adverse cardiovascular events. PNI provided modest incremental prognostic information and may serve as a complementary marker for early risk stratification.

## 1 Introduction

Acute heart failure (AHF) is a common and clinically heterogeneous syndrome encountered in emergency departments and is characterized by rapid onset or worsening of symptoms and signs of cardiac dysfunction (1). It remains a major cause of unplanned hospitalization, short-term clinical deterioration, intensive care utilization, and early post-discharge adverse events (2). Because patients with AHF may present with diverse hemodynamic profiles, variable degrees of congestion, respiratory compromise, renal dysfunction, and myocardial injury, early risk stratification is essential for guiding treatment intensity, monitoring requirements, and disposition decisions (3, 4). Contemporary studies have emphasized that risk assessment in AHF should integrate clinical presentation, vital signs, cardiac biomarkers, renal function, imaging findings, and treatment response rather than relying on a single parameter (5–7). Malnutrition and systemic inflammation are increasingly recognized as important contributors to adverse outcomes in patients with heart failure (8, 9). Nutritional impairment in heart failure may arise from reduced dietary intake, intestinal edema, hepatic congestion, renal dysfunction, systemic inflammation, catabolic activation, and recurrent hospitalization (10). These abnormalities may reduce physiological reserve and impair recovery during acute decompensation. In addition, albumin-based indices and nutrition-related risk scores have shown prognostic relevance in patients with heart failure, supporting the clinical value of routine nutritional assessment in this population (11, 12). However, nutritional risk is still not systematically incorporated into emergency risk evaluation for AHF.

The prognostic nutritional index (PNI), calculated from serum albumin concentration and peripheral lymphocyte count, is a simple composite indicator reflecting nutritional reserve and immune-inflammatory status. Unlike complex nutritional assessment tools, PNI can be derived from routine laboratory tests obtained at admission and is therefore suitable for emergency and inpatient settings. Recent evidence suggests that lower PNI is associated with adverse outcomes in patients with heart failure, including higher mortality and rehospitalization risk (13). In patients with acute decompensated heart failure, PNI has also been evaluated in combination with electrolyte abnormalities, further indicating its potential role in identifying patients with poor prognosis (14). Population-based studies have also shown associations between PNI and cardiovascular mortality or heart failure prevalence, suggesting that PNI may capture systemic vulnerability beyond traditional cardiovascular risk factors (15, 16).

Previous studies have associated a lower PNI with mortality and heart failure hospitalization; however, most have focused on longer-term outcomes or selected populations (15, 17). Evidence remains limited regarding whether PNI measured during the early assessment of patients with AHF in the emergency department provides incremental prognostic information beyond conventional clinical variables for adverse cardiovascular events occurring after PNI measurement. To address this gap, we conducted a retrospective cohort study with an explicitly defined index PNI time and restricted outcome ascertainment to subsequent in-hospital events. We evaluated the association between early PNI and incident in-hospital adverse cardiovascular events and assessed whether adding PNI to a conventional clinical model improved prognostic performance.

## 2 Methods

### 2.1 Study design

This retrospective cohort study was conducted in the emergency department of the study institution and included the medical records of consecutive adult patients presenting with AHF between March 2022 and March 2026. All examinations and treatments were performed as part of routine clinical care, and no diagnostic or therapeutic intervention was assigned by the study protocol. The study aimed to evaluate the association between the PNI, calculated from serum albumin and peripheral lymphocyte measurements obtained early after admission, and a predefined composite of in-hospital adverse cardiovascular events, as well as the incremental prognostic information provided by PNI beyond conventional clinical variables. The index PNI time was defined as the later collection time of the serum albumin and peripheral lymphocyte specimens used to calculate PNI. Clinical variables available at or before the index PNI time were retrospectively extracted according to predefined time windows, whereas incident outcomes were ascertained strictly after the index PNI time until hospital discharge or in-hospital death. The study protocol was approved by the institutional ethics committee. Given the retrospective nature of this study, the requirement for written informed consent was waived by the committee. The study was conducted in accordance with the Declaration of Helsinki and applicable institutional requirements, and all patient data were anonymized before analysis.

### 2.2 Eligibility criteria

Patients were eligible if they met all of the following criteria: (1) age ≥18 years; (2) presentation to the emergency department during the study period with new or worsening symptoms or signs compatible with AHF; (3) a diagnosis of AHF documented during the index hospitalization; (4) availability of serum albumin and peripheral lymphocyte measurements obtained within 24 h after admission, allowing calculation of the PNI; and (5) sufficient inpatient records to determine the index PNI time and ascertain the primary outcome until hospital discharge or in-hospital death. Missing data for covariates other than the PNI components and study outcomes did not preclude inclusion. Both new-onset AHF and acute decompensation of chronic heart failure were eligible. For study inclusion, AHF was retrospectively confirmed by new or worsening symptoms or signs of heart failure together with at least one objective finding obtained no later than the index PNI time: (1) an NT-proBNP concentration measured within 24 h after admission that met the prespecified age-specific rule-in threshold (≥450 pg/mL for patients aged < 50 years, ≥900 pg/mL for those aged 50–75 years, or ≥1,800 pg/mL for those aged >75 years); (2) echocardiographic evidence of a clinically relevant structural or functional cardiac abnormality consistent with heart failure; or (3) chest radiographic or computed tomographic evidence of pulmonary vascular congestion or pulmonary edema attributable to heart failure. The presentation was not more plausibly explained by an alternative non-cardiac condition. No specific left ventricular ejection fraction threshold was required for inclusion. Patients were excluded if they met any of the following criteria: (1) missing serum albumin or peripheral lymphocyte measurements that precluded PNI calculation, or insufficient records to ascertain the primary outcome; (2) severe acute infection or sepsis, active malignancy, severe hepatic failure, or a hematologic disorder likely to substantially affect serum albumin concentration or lymphocyte count; (3) albumin infusion or systemic immunosuppressive therapy before collection of the specimens used to calculate PNI; (4) admission primarily for a non-cardiogenic condition, including trauma, poisoning, acute pulmonary embolism, or an acute cerebrovascular event; or (5) discharge against medical advice or transfer shortly after emergency presentation that precluded adequate post-index outcome ascertainment.

### 2.3 Data collection and quality control

Clinical data were retrospectively abstracted from electronic medical records using a standardized form. Baseline variables included age, sex, body mass index, smoking and alcohol use, hypertension, diabetes mellitus, coronary artery disease, atrial fibrillation, chronic kidney disease, chronic obstructive pulmonary disease, and previous heart failure hospitalization. Pre-admission use of diuretics, β-blockers, renin–angiotensin system inhibitors, mineralocorticoid receptor antagonists, sodium–glucose cotransporter 2 inhibitors, antiplatelet agents, anticoagulants, and statins was also recorded. Clinical variables included blood pressure, heart rate, respiratory rate, peripheral oxygen saturation, body temperature, Killip class, New York Heart Association functional class, pulmonary rales, peripheral edema, jugular venous distension, hypotension, and hypoperfusion. The measurement closest to and at or before the index PNI time within the preceding 6 h was used; if unavailable, the closest earlier measurement recorded after admission was selected. Hypotension was defined as systolic blood pressure < 90 mmHg or vasopressor requirement to maintain systolic blood pressure ≥90 mmHg after adequate volume filling. Hypoperfusion was defined by cool extremities, altered mental status, urine output < 0.5 mL/kg/h, or serum lactate >2.0 mmol/L. Noninvasive ventilation, invasive mechanical ventilation, continuous vasoactive support, and ICU or equivalent critical-care location were classified as baseline support only when ongoing at the index PNI time. Advanced support was defined as the presence of at least one of these measures. Indeterminate timing was recorded as missing, whereas initiation or reinitiation strictly after the index time was evaluated as a post-index outcome.

Laboratory variables included blood cell counts, serum albumin, total protein, C-reactive protein, serum creatinine, estimated glomerular filtration rate (eGFR), serum sodium, high-sensitivity cardiac troponin I, creatine kinase–myocardial band mass, N-terminal pro-B-type natriuretic peptide (NT-proBNP), liver function indices, prothrombin time, and international normalized ratio. The first eligible result obtained within 24 h after admission and at or before the index PNI time was abstracted. NT-proBNP was natural-log transformed for multivariable analyses, and eGFR was calculated using the 2021 Chronic Kidney Disease Epidemiology Collaboration creatinine equation. Echocardiographic variables included left ventricular ejection fraction (LVEF), left atrial diameter, left ventricular end-diastolic diameter, tricuspid annular plane systolic excursion, right ventricular dysfunction, and estimated pulmonary artery systolic pressure. The examination closest to and at or before the index PNI time was preferentially used. If no pre-index LVEF was available, the first measurement obtained within 24 h after the index time and before any primary outcome event was accepted; otherwise, LVEF was recorded as missing. LVEF was categorized as < 40%, 40%−49%, or ≥50%. Right ventricular dysfunction was defined as tricuspid annular plane systolic excursion < 17 mm and/or documented right ventricular systolic dysfunction. Chest imaging variables were abstracted from the eligible radiograph or computed tomography report closest to and at or before the index PNI time. If unavailable, the first examination completed within 24 h after the index time and before any primary outcome event was used. Pulmonary congestion, pleural effusion, and pulmonary edema were recorded. Except for LVEF, echocardiographic and chest imaging variables were used only for descriptive analyses.

Variable definitions and abstraction windows were specified before construction of the final analytical database. Data were abstracted by a trained investigator and independently verified against the source records by a second investigator, with specific verification of PNI components, index timestamps, and outcomes. Ambiguous outcomes were independently reviewed by two clinicians without access to PNI-group assignment. Disagreements were resolved by consensus or adjudication by a third senior clinician. Range, consistency, duplicate-record, and temporal-order checks were completed before analysis.

### 2.4 Definition of exposure variable and patient grouping

The PNI was the primary exposure and was calculated as follows: PNI = serum albumin (g/L) + 5 × peripheral lymphocyte count (10^9^/L). The first eligible serum albumin and peripheral lymphocyte measurements obtained within 24 h after admission were used. If the specimens were collected at different times, the later collection time was defined as the index PNI time. Based on a literature-derived threshold specified before data analysis, patients were classified into a low-PNI group (PNI < 45) or a high-PNI group (PNI ≥45) (18, 19).

### 2.5 Outcome definitions

The primary outcome was a composite of incident adverse cardiovascular events occurring strictly after the index PNI time and before hospital discharge or in-hospital death. It was defined as the first occurrence of all-cause death, cardiogenic shock, sustained ventricular tachycardia or ventricular fibrillation, cardiac arrest requiring cardiopulmonary resuscitation, acute myocardial infarction, or worsening AHF requiring treatment escalation. Cardiogenic shock was defined as systolic blood pressure < 90 mmHg for at least 30 min or vasopressor requirement to maintain systolic blood pressure ≥90 mmHg, together with clinical or biochemical evidence of hypoperfusion attributable primarily to cardiac dysfunction. Shock present at the index assessment was treated as a baseline characteristic unless it subsequently resolved and recurred. Sustained ventricular tachycardia was defined as an episode lasting ≥30 s or requiring earlier termination because of hemodynamic compromise; any documented ventricular fibrillation was included. Cardiac arrest was defined as loss of effective circulation requiring chest compressions and/or defibrillation. Acute myocardial infarction required a rise and/or fall in cardiac troponin with at least one value above the assay-specific 99th-percentile upper reference limit, accompanied by evidence of acute myocardial ischemia. Acute myocardial injury without ischemic evidence was excluded. Worsening AHF was defined as new or worsening congestion or hypoperfusion after the index assessment requiring escalation beyond treatment already present at index. Qualifying escalation included intensified intravenous diuretic therapy, addition of a second diuretic, initiation of an intravenous vasodilator, inotrope, or vasopressor, new ventilatory or mechanical circulatory support, or ICU transfer. ICU admission or treatment escalation alone was not considered a primary component unless the clinical criteria for worsening AHF were met.

Secondary outcomes included the individual components of the primary endpoint, cardiovascular death, post-index ICU admission, mechanical ventilation, continuous vasoactive support, length of hospital stay, and discharge status. Cardiovascular death was defined as death attributed to AHF, cardiogenic shock, acute myocardial infarction, fatal ventricular arrhythmia, sudden cardiac death, or another documented cardiovascular cause. It was treated as a subset of all-cause death and not as an additional primary component. ICU admission was a secondary outcome and was not used as a subgroup variable. Support already ongoing at the index assessment was not counted as an incident outcome unless it was discontinued and subsequently reinitiated. Mechanical ventilation comprised new noninvasive or invasive ventilation after index and was counted once per patient, although the two modalities were also reported separately. Length of stay was calculated from admission to discharge or in-hospital death using elapsed hours divided by 24. Discharge status was categorized as discharge home alive, discharge to a rehabilitation or nursing facility, transfer to another acute-care hospital, or in-hospital death. For patients with multiple component events, the composite endpoint was counted once, and the first qualifying event established the temporal sequence; individual components were not mutually exclusive. Outcomes were retrospectively ascertained from the integrated electronic medical record, including clinical documentation, vital signs, medication records, laboratory and imaging examinations, electrocardiography or telemetry, respiratory and procedure records, ICU transfer logs, resuscitation records, and discharge summaries. The hard clinical composite was defined as all-cause death, cardiogenic shock, sustained ventricular tachycardia or ventricular fibrillation, cardiac arrest requiring cardiopulmonary resuscitation, or acute myocardial infarction; worsening AHF and resource-utilization outcomes were excluded.

### 2.6 Statistical analysis

All statistical analyses were performed using IBM SPSS Statistics, version 30.0 (IBM Corp., Armonk, NY, USA). Continuous variables were summarized as mean ± standard deviation or median (interquartile range), and categorical variables as n/N (%). Groups were compared using Welch's *t* test, Mann–Whitney U test, or Pearson's chi-square test, as appropriate. Missing covariates were handled using multiple imputation by chained equations with 20 datasets. Predictive mean matching and logistic or multinomial regression were used for continuous and categorical data. Serum creatinine was imputed before passive recalculation of eGFR. PNI, its components, and outcomes were not imputed. Estimates were pooled using Rubin's rules. Binary logistic regression estimated odds ratios (ORs) and 95% confidence intervals (CIs) for the primary outcome. PNI was modeled as < 45 vs. ≥45 and continuously. Model 1 was unadjusted; Model 2 adjusted for age, sex, and body mass index; Model 3 additionally adjusted for diabetes, chronic kidney disease, and previous HF hospitalization; and Model 4 further adjusted for systolic blood pressure, heart rate, LVEF, ln(NT-proBNP), serum sodium, and advanced support. Continuous-PNI estimates were rescaled per 5- and 10-point increase. Discrimination of PNI alone, the conventional clinical model, and the model with PNI added was assessed using receiver operating characteristic curves and area under the curve (AUC). Cutoffs maximized the Youden index. Calibration was assessed using the Hosmer–Lemeshow test, calibration intercept and slope, and Brier score. Incremental value was evaluated using ΔAUC, net reclassification improvement, and integrated discrimination improvement. The cohort-derived PNI cutoff was exploratory and did not replace the literature-derived cutoff of 45. Confidence intervals and optimism-corrected performance were obtained using 1,000 bootstrap resamples. Subgroup analyses evaluated interactions for age, sex, LVEF, diabetes, chronic kidney disease, NT-proBNP, and atrial fibrillation. Sensitivity analyses excluded baseline component events, used complete cases or the hard clinical composite, excluded CRP ≥50 mg/L, and modeled PNI quartiles with a trend test. All tests were two-sided, with *P* < 0.05 considered significant.

## 3 Results

### 3.1 Patient selection and study population

A total of 398 adult patients with a documented diagnosis of AHF following presentation to the emergency department between March 2022 and March 2026 were retrospectively screened. After medical record review, 31 patients were excluded: 9 had missing serum albumin or peripheral lymphocyte measurements that precluded calculation of the PNI, or insufficient records for primary-outcome ascertainment; 6 had severe acute infection or sepsis; 5 had active malignancy, severe hepatic failure, or a hematologic disorder; 4 had received albumin infusion or systemic immunosuppressive therapy before collection of the specimens used to calculate PNI; 5 were admitted primarily for a non-cardiogenic condition; and 2 were discharged against medical advice or transferred shortly after emergency presentation, precluding adequate post-index outcome ascertainment. The remaining 367 patients met the prespecified AHF confirmation criteria and all other eligibility criteria. Based on the literature-derived PNI cutoff of 45, 156 patients (42.5%) were classified into the low-PNI group (PNI < 45) and 211 (57.5%) into the high-PNI group (PNI ≥45).

### 3.2 Baseline demographic and clinical characteristics

Among the 367 included patients, 156 were classified into the low-PNI group and 211 into the high-PNI group. Patients in the low-PNI group were older than those in the high-PNI group (74.2 ± 10.6 vs. 69.1 ± 11.3 years, *P* < 0.001) and had a lower body mass index (22.6 ± 3.2 vs. 24.1 ± 3.5 kg/m^2^, *P* < 0.001). Chronic kidney disease (39.7% vs. 24.2%, *P* = 0.001) and previous hospitalization for heart failure (50.6% vs. 37.0%, *P* = 0.009) were also more frequent in the low-PNI group. Sex, smoking status, alcohol consumption, other recorded comorbidities, and pre-admission cardiovascular medications did not differ significantly between the groups (Table 1).

**Table 1:** Baseline demographic and clinical characteristics according to PNI group.

Variable	Low-PNI group (*n* = 156)	High-PNI group (*n* = 211)	Statistic	*P*-value
Age, years	74.2 ± 10.6	69.1 ± 11.3	t = 4.388	< 0.001
Male sex	88/156 (56.4)	128/211 (60.7)	χ^2^ = 0.670	0.413
Body mass index, kg/m^2^	22.6 ± 3.2 (*n* = 152)	24.1 ± 3.5 (*n* = 206)	t = −4.212	< 0.001
Current smoking	58/153 (37.9)	72/207 (34.8)	χ^2^ = 0.373	0.542
Alcohol consumption	36/151 (23.8)	50/205 (24.4)	χ^2^ = 0.014	0.905
Hypertension	118/156 (75.6)	148/211 (70.1)	χ^2^ = 1.360	0.244
Diabetes mellitus	67/156 (42.9)	70/211 (33.2)	χ^2^ = 3.662	0.056
Coronary artery disease	89/156 (57.1)	101/211 (47.9)	χ^2^ = 3.030	0.082
Atrial fibrillation	76/156 (48.7)	84/211 (39.8)	χ^2^ = 2.894	0.089
Chronic kidney disease	62/156 (39.7)	51/211 (24.2)	χ^2^ = 10.207	0.001
Chronic obstructive pulmonary disease	31/156 (19.9)	28/211 (13.3)	χ^2^ = 2.897	0.089
Previous hospitalization for heart failure	79/156 (50.6)	78/211 (37.0)	χ^2^ = 6.851	0.009
Pre-admission medications				
Diuretics	103/153 (67.3)	123/207 (59.4)	χ^2^ = 2.350	0.125
β-blockers	72/153 (47.1)	109/207 (52.7)	χ^2^ = 1.103	0.294
ACE inhibitors	24/153 (15.7)	42/207 (20.3)	χ^2^ = 1.245	0.264
Angiotensin receptor blockers	46/153 (30.1)	73/207 (35.3)	χ^2^ = 1.075	0.300
Angiotensin receptor–neprilysin inhibitors	33/153 (21.6)	61/207 (29.5)	χ^2^ = 2.846	0.092
Mineralocorticoid receptor antagonists	70/153 (45.8)	86/207 (41.5)	χ^2^ = 0.634	0.426
Sodium–glucose cotransporter 2 inhibitors	39/153 (25.5)	68/207 (32.9)	χ^2^ = 2.282	0.131
Antiplatelet agents	74/153 (48.4)	93/207 (44.9)	χ^2^ = 0.418	0.518
Anticoagulants	58/153 (37.9)	75/207 (36.2)	χ^2^ = 0.106	0.745
Statins	83/153 (54.2)	108/207 (52.2)	χ^2^ = 0.152	0.697

ACE, angiotensin-converting enzyme; BMI, body mass index; PNI, prognostic nutritional index.

### 3.3 Clinical status at index PNI assessment

At the index PNI assessment, the low-PNI group had lower systolic blood pressure (118.6 ± 23.4 vs. 126.8 ± 21.7 mmHg, *P* = 0.001) and peripheral oxygen saturation (91.8 ± 5.9% vs. 94.3 ± 4.8%, *P* < 0.001), together with higher heart rate (106.3 ± 21.5 vs. 99.7 ± 19.6 beats/min, *P* = 0.003) and respiratory rate (24.8 ± 5.1 vs. 22.7 ± 4.6 breaths/min, *P* < 0.001). Killip class III–IV (71.2% vs. 53.1%, *P* < 0.001), New York Heart Association class IV (47.4% vs. 31.3%, *P* = 0.002), hypotension (26.9% vs. 16.1%, *P* = 0.012), and hypoperfusion (25.0% vs. 13.7%, *P* = 0.006) were more frequent in the low-PNI group. Among patients with definitive treatment-timing information, noninvasive ventilation, vasoactive support, and critical-care or intensive care unit location were more frequently present at the index assessment in the low-PNI group, whereas the difference in invasive mechanical ventilation was not statistically significant (Table 2).

**Table 2:** Clinical status at index PNI assessment.

Variable	Low-PNI group (*n* = 156)	High-PNI group (*n* = 211)	Statistic	*P*-value
Systolic blood pressure, mmHg	118.6 ± 23.4 (*n* = 155)	126.8 ± 21.7 (*n* = 210)	t = −3.412	0.001
Diastolic blood pressure, mmHg	70.8 ± 14.2 (*n* = 155)	74.9 ± 13.6 (*n* = 210)	t = −2.776	0.006
Heart rate, beats/min	106.3 ± 21.5 (*n* = 155)	99.7 ± 19.6 (*n* = 210)	t = 3.009	0.003
Respiratory rate, breaths/min	24.8 ± 5.1 (*n* = 156)	22.7 ± 4.6 (*n* = 211)	t = 4.064	< 0.001
Peripheral oxygen saturation, %	91.8 ± 5.9 (*n* = 154)	94.3 ± 4.8 (*n* = 209)	t = −4.337	< 0.001
Body temperature, °C	36.7 ± 0.4 (*n* = 156)	36.7 ± 0.4 (*n* = 211)	t = 0.684	0.495
Killip class III–IV	111/156 (71.2)	112/211 (53.1)	χ^2^ = 12.288	< 0.001
NYHA class IV	74/156 (47.4)	66/211 (31.3)	χ^2^ = 9.922	0.002
Pulmonary rales	128/156 (82.1)	152/211 (72.0)	χ^2^ = 4.972	0.026
Peripheral edema	96/156 (61.5)	106/211 (50.2)	χ^2^ = 4.629	0.031
Jugular venous distension	67/156 (42.9)	68/211 (32.2)	χ^2^ = 4.433	0.035
Hypotension	42/156 (26.9)	34/211 (16.1)	χ^2^ = 6.382	0.012
Hypoperfusion	39/156 (25.0)	29/211 (13.7)	χ^2^ = 7.528	0.006
Advanced support ongoing at index PNI assessment				
Noninvasive ventilation	40/151 (26.5)	36/204 (17.6)	χ^2^ = 4.033	0.045
Invasive mechanical ventilation	10/151 (6.6)	7/204 (3.4)	χ^2^ = 1.938	0.164
Vasoactive drug support	18/151 (11.9)	12/204 (5.9)	χ^2^ = 4.089	0.043
Critical-care/ICU location	17/151 (11.3)	11/204 (5.4)	χ^2^ = 4.110	0.043

ICU, intensive care unit; NYHA, New York Heart Association; PNI, prognostic nutritional index.

### 3.4 Laboratory parameters at index PNI assessment

As expected from the PNI formula, the low-PNI group had lower lymphocyte counts (0.9 ± 0.3 vs. 1.6 ± 0.4 × 10^9^/L, *P* < 0.001) and serum albumin concentrations (33.1 ± 3.8 vs. 39.8 ± 3.3 g/L, *P* < 0.001). Patients with low PNI also had higher C-reactive protein levels (22.6 [10.0–39.3] vs. 9.8 [5.8–19.6] mg/L, *P* < 0.001), serum creatinine (122 ± 52 vs. 94 ± 39 μmol/L, *P* < 0.001), high-sensitivity cardiac troponin I (57 [29–162] vs. 35 [18–88] ng/L, *P* < 0.001), and N-terminal pro-B-type natriuretic peptide concentrations (7,912 [4,107–14,865] vs. 5,305 [2,658–9,741] pg/mL, *P* < 0.001). The low-PNI group had lower estimated glomerular filtration rate (54.7 ± 21.1 vs. 68.1 ± 22.2 mL/min/1.73 m^2^, *P* < 0.001) and serum sodium concentration (137.1 ± 4.6 vs. 138.4 ± 3.8 mmol/L, *P* = 0.005) (Table 3).

**Table 3:** Laboratory parameters at index PNI assessment.

Variable	Low-PNI group (*n* = 156)	High-PNI group (*n* = 211)	Statistic	*P-*value
White blood cell count, × 10^9^/L	8.7 ± 2.9 (*n* = 156)	8.4 ± 2.5 (*n* = 211)	t = 1.038	0.300
Neutrophils, × 10^9^/L	6.9 ± 3.0 (*n* = 156)	6.0 ± 2.4 (*n* = 211)	t = 3.087	0.002
Lymphocytes, × 10^9^/L	0.9 ± 0.3 (*n* = 156)	1.6 ± 0.4 (*n* = 211)	t = −19.157	< 0.001
Hemoglobin, g/L	120.9 ± 20.2 (*n* = 156)	129.9 ± 16.7 (*n* = 211)	t = −4.536	< 0.001
Platelets, × 10^9^/L	208.7 ± 74.8 (*n* = 156)	218.9 ± 69.7 (*n* = 211)	t = −1.329	0.185
Albumin, g/L	33.1 ± 3.8 (*n* = 156)	39.8 ± 3.3 (*n* = 211)	t = −17.645	< 0.001
Total protein, g/L	62.8 ± 7.5 (*n* = 156)	68.2 ± 6.9 (*n* = 211)	t = −7.053	< 0.001
C-reactive protein, mg/L	22.6 (10.0–39.3) (*n* = 142)	9.8 (5.8–19.6) (*n* = 197)	U = 22,560	< 0.001
Serum creatinine, μmol/L	122 ± 52 (*n* = 154)	94 ± 39 (*n* = 210)	t = 5.622	< 0.001
eGFR, mL/min/1.73 m^2^	54.7 ± 21.1 (*n* = 154)	68.1 ± 22.2 (*n* = 210)	t = −5.855	< 0.001
Serum sodium, mmol/L	137.1 ± 4.6 (*n* = 153)	138.4 ± 3.8 (*n* = 210)	t = −2.857	0.005
High-sensitivity cardiac troponin I, ng/L	57 (29–162) (*n* = 152)	35 (18–88) (*n* = 205)	U = 20,075	< 0.001
CK-MB mass, ng/mL	5.382 (3.007–9.416) (*n* = 156)	4.104 (2.532–7.673) (*n* = 211)	U = 18,849	0.017
NT-proBNP, pg/mL	7,912 (4,107–14,865) (*n* = 155)	5,305 (2,658–9,741) (*n* = 210)	U = 21,651	< 0.001
Alanine aminotransferase, U/L	31 (19–59) (*n* = 156)	27 (18–45) (*n* = 211)	U = 18,501	0.040
Aspartate aminotransferase, U/L	36 (24–67) (*n* = 156)	30 (22–49) (*n* = 211)	U = 18,969	0.014
Total bilirubin, μmol/L	18.9 (12.7–29.4) (*n* = 156)	16.1 (11.2–23.7) (*n* = 211)	U = 18,909	0.016
Prothrombin time, s	14.2 ± 2.5 (*n* = 156)	13.6 ± 2.1 (*n* = 211)	t = 2.430	0.016
International normalized ratio	1.19 ± 0.22 (*n* = 156)	1.14 ± 0.18 (*n* = 211)	t = 2.322	0.021

CK-MB, creatine kinase–myocardial band; CKD-EPI, Chronic Kidney Disease Epidemiology Collaboration; CMIA, chemiluminescent microparticle immunoassay; eGFR, estimated glomerular filtration rate; hs-cTnI, high-sensitivity cardiac troponin I; PNI, prognostic nutritional index; Scr, serum creatinine; SD, standard deviation.

### 3.5 Echocardiographic and chest imaging findings

The low-PNI group had a lower mean left ventricular ejection fraction (41.8 ± 12.4% vs. 45.6 ± 11.9%, *P* = 0.004), larger left atrial and left ventricular end-diastolic dimensions, and lower tricuspid annular plane systolic excursion. Right ventricular dysfunction was documented in 48.1% of evaluable patients in the low-PNI group and 31.5% in the high-PNI group (*P* = 0.003). Pulmonary congestion (80.5% vs. 66.8%, *P* = 0.004), pleural effusion (53.9% vs. 40.9%, *P* = 0.014), and pulmonary edema (46.8% vs. 30.8%, *P* = 0.002) were also more frequent in the low-PNI group (Table 4).

**Table 4:** Echocardiographic and chest imaging findings.

Variable	Low-PNI group (*n* = 156)	High-PNI group (*n* = 211)	Statistic	*P*-value
Left ventricular ejection fraction, %	41.8 ± 12.4 (*n* = 147)	45.6 ± 11.9 (*n* = 202)	t = −2.875	0.004
Left ventricular ejection fraction < 40%	66/147 (44.9)	70/202 (34.7)	χ^2^ = 3.755	0.053
Left atrial diameter, mm	45.2 ± 7.9 (*n* = 147)	43.1 ± 7.4 (*n* = 202)	t = 2.518	0.012
Left ventricular end-diastolic diameter, mm	58.6 ± 8.7 (*n* = 147)	56.2 ± 8.1 (*n* = 202)	t = 2.619	0.009
Tricuspid annular plane systolic excursion, mm	16.8 ± 4.1 (*n* = 129)	18.1 ± 3.8 (*n* = 181)	t = −2.836	0.005
Right ventricular dysfunction	62/129 (48.1)	57/181 (31.5)	χ^2^ = 8.744	0.003
Estimated pulmonary artery systolic pressure, mmHg	43.5 ± 13.4 (*n* = 125)	39.1 ± 12.1 (*n* = 178)	t = 2.927	0.003
Pulmonary congestion on chest imaging	124/154 (80.5)	139/208 (66.8)	χ^2^ = 8.350	0.004
Pleural effusion on chest imaging	83/154 (53.9)	85/208 (40.9)	χ^2^ = 6.041	0.014
Pulmonary edema on chest imaging	72/154 (46.8)	64/208 (30.8)	χ^2^ = 9.639	0.002

CT, computed tomography; LVEDD, left ventricular end-diastolic diameter; LVEF, left ventricular ejection fraction; PASP, pulmonary artery systolic pressure; PNI, prognostic nutritional index; TAPSE, tricuspid annular plane systolic excursion; TR, tricuspid regurgitation.

### 3.6 Primary and secondary in-hospital outcomes

The outcome window extended strictly from the index PNI assessment to hospital discharge or in-hospital death. The primary composite endpoint was recorded once per patient, whereas its component events were not mutually exclusive. Cardiovascular death and post-index intensive care unit admission were classified as secondary outcomes rather than separate primary components; detailed operational definitions are provided in Supplementary Table S1. Overall, 112 patients (30.5%) experienced the primary composite endpoint. Its incidence was higher in the low-PNI group than in the high-PNI group (43.6% vs. 20.9%, *P* < 0.001). The low-PNI group had higher incidences of all-cause death (14.7% vs. 5.7%, *P* = 0.004), cardiogenic shock (18.6% vs. 8.5%, *P* = 0.004), sustained ventricular tachycardia or ventricular fibrillation (9.6% vs. 3.8%, *P* = 0.023), cardiac arrest requiring cardiopulmonary resuscitation (7.7% vs. 2.8%, *P* = 0.033), and worsening AHF requiring treatment escalation (32.7% vs. 15.2%, *P* < 0.001). The incidence of acute myocardial infarction did not differ significantly between the groups (6.4% vs. 3.3%, *P* = 0.163). Post-index intensive care unit admission (22.4% vs. 10.4%, *P* = 0.002), mechanical ventilation (25.0% vs. 11.8%, *P* = 0.001), and vasoactive support (26.9% vs. 13.3%, *P* = 0.001) were more frequent in the low-PNI group. Median length of stay was longer in the low-PNI group (10 [7–14] vs. 8 [6–11] days, *P* < 0.001), and discharge status differed between the groups overall (*P* = 0.005) (Table 5).

**Table 5:** Primary and secondary in-hospital outcomes.

Outcome	Low-PNI group, n/N (%)	High-PNI group, n/N (%)	Statistic	*P*-value
Primary composite endpoint	68/156 (43.6)	44/211 (20.9)	χ^2^ = 21.866	< 0.001
All-cause death	23/156 (14.7)	12/211 (5.7)	χ^2^ = 8.527	0.004
Cardiogenic shock	29/156 (18.6)	18/211 (8.5)	χ^2^ = 8.127	0.004
Sustained VT/VF	15/156 (9.6)	8/211 (3.8)	χ^2^ = 5.179	0.023
Cardiac arrest requiring CPR	12/156 (7.7)	6/211 (2.8)	χ^2^ = 4.521	0.033
Acute myocardial infarction	10/156 (6.4)	7/211 (3.3)	χ^2^ = 1.942	0.163
Worsening AHF requiring treatment escalation	51/156 (32.7)	32/211 (15.2)	χ^2^ = 15.742	< 0.001
Cardiovascular death	20/156 (12.8)	10/211 (4.7)	χ^2^ = 7.803	0.005
ICU admission after PNI assessment	35/156 (22.4)	22/211 (10.4)	χ^2^ = 9.860	0.002
Any mechanical ventilation after PNI assessment	39/156 (25.0)	25/211 (11.8)	χ^2^ = 10.775	0.001
Noninvasive ventilation	31/156 (19.9)	20/211 (9.5)	χ^2^ = 8.097	0.004
Invasive mechanical ventilation	12/156 (7.7)	7/211 (3.3)	χ^2^ = 3.497	0.061
Vasoactive support after PNI assessment	42/156 (26.9)	28/211 (13.3)	χ^2^ = 10.831	0.001
Length of stay, days	10 (7–14)	8 (6–11)	U = 21,370	< 0.001
Discharge status, overall	—	—	χ^2^ = 12.780	0.005
Discharged home alive	110/156 (70.5)	180/211 (85.3)	—	—
Rehabilitation/nursing facility	12/156 (7.7)	10/211 (4.7)	—	—
Transferred to another acute-care hospital	11/156 (7.1)	9/211 (4.3)	—	—
In-hospital death	23/156 (14.7)	12/211 (5.7)	—	—

AHF, acute heart failure; CPR, cardiopulmonary resuscitation; ICU, intensive care unit; PNI, prognostic nutritional index; VF, ventricular fibrillation; VT, ventricular tachycardia.

### 3.7 Association between PNI and the primary outcome

In the unadjusted analysis, low PNI was associated with higher odds of the primary composite endpoint compared with high PNI (OR = 2.93, 95% CI: 1.85–4.64, *P* < 0.001). The association remained after adjustment for demographic characteristics, comorbidities, clinical measurements, cardiac function, laboratory indicators, and advanced support ongoing at the index assessment (adjusted OR = 1.92, 95% CI: 1.10–3.35, *P* = 0.022). When analyzed continuously, each 1-point increase in PNI was associated with lower odds of the primary outcome in the fully adjusted model (adjusted OR = 0.93, 95% CI: 0.89–0.98, *P* = 0.003). The corresponding ORs were 0.70 (95% CI: 0.56–0.90) per 5-point increase and 0.49 (95% CI: 0.31–0.82) per 10-point increase. These scaled estimates were derived from the same continuous PNI coefficient (Table 6).

**Table 6:** Association between PNI and the primary outcome.

Exposure/model	Patients/events	OR	95% CI	Wald χ^2^	*P*-value
Low PNI (<45) vs. high PNI (≥45; reference)					
Model 1: unadjusted	367/112	2.93	1.85–4.64	21.13	< 0.001
Model 2: demographic adjustment	367/112	2.54	1.55–4.16	13.70	< 0.001
Model 3: demographic + comorbidity adjustment	367/112	2.22	1.32–3.73	9.06	0.003
Model 4: fully adjusted	367/112	1.92	1.10–3.35	5.27	0.022
PNI as a continuous exposure, per 1-point increase					
Model 1: unadjusted	367/112	0.88	0.84–0.92	30.34	< 0.001
Model 2: demographic adjustment	367/112	0.90	0.86–0.94	21.56	< 0.001
Model 3: demographic + comorbidity adjustment	367/112	0.91	0.87–0.96	14.10	< 0.001
Model 4: fully adjusted	367/112	0.93	0.89–0.98	8.72	0.003
Clinically scaled continuous PNI in Model 4					
Per 5-point increase	367/112	0.70	0.56–0.90	8.72	0.003
Per 10-point increase	367/112	0.49	0.31–0.82	8.72	0.003

CI, confidence interval; LVEF, left ventricular ejection fraction; NT-proBNP, N-terminal pro-B-type natriuretic peptide; OR, odds ratio; PNI, prognostic nutritional index.

### 3.8 Predictive performance and incremental value of PNI

PNI alone showed moderate apparent discrimination for the primary outcome, with an AUC of 0.714 (95% CI: 0.658–0.771). The exploratory ROC-derived cutoff was 44.8, with a sensitivity of 60.7% and specificity of 65.5%. The conventional clinical model had an AUC of 0.776 (95% CI: 0.726–0.826), whereas the model incorporating continuous PNI had an AUC of 0.829 (95% CI: 0.786–0.872), with a sensitivity of 76.8% and specificity of 74.1% at its ROC-derived probability cutoff. The Hosmer–Lemeshow *P*-value for the combined model was 0.627 (Table 7). Compared with the conventional model, the combined model had an AUC increment of 0.053 (95% CI: 0.021–0.085, *P* = 0.001), a net reclassification improvement of 0.287 (95% CI: 0.126–0.448, *P* < 0.001), and an integrated discrimination improvement of 0.061 (95% CI: 0.034–0.088, *P* < 0.001) (Supplementary Table S2). After bootstrap correction, the AUCs were 0.752 for the conventional model and 0.803 for the combined model. The corresponding corrected calibration slopes were 0.86 and 0.88, and the corrected Brier scores were 0.174 and 0.151, respectively (Supplementary Table S3).

**Table 7:** Predictive performance for the primary outcome.

Model	Patients/events	ROC-derived cutoff	AUC	95% CI	Sensitivity, %	Specificity, %	Youden index	Hosmer–Lemeshow χ^2^	Hosmer–Lemeshow *P*-value
PNI alone	367/112	PNI 44.8	0.714	0.658–0.771	60.7	65.5	0.262	5.84	0.665
Conventional clinical model	367/112	Predicted risk 0.292	0.776	0.726–0.826	71.4	70.2	0.416	7.42	0.492
Conventional model + PNI	367/112	Predicted risk 0.304	0.829	0.786–0.872	76.8	74.1	0.509	6.18	0.627

AUC, area under the receiver operating characteristic curve; CI, confidence interval; eGFR, estimated glomerular filtration rate; LVEF, left ventricular ejection fraction; NT-proBNP, N-terminal pro-B-type natriuretic peptide; PNI, prognostic nutritional index; ROC, receiver operating characteristic.

### 3.9 Subgroup analyses

Across the seven baseline-defined subgroup variables, all point estimates favored higher odds of the primary outcome in the low-PNI group, with adjusted ORs ranging from 1.78 to 2.10. However, several subgroup-specific confidence intervals included 1.00, reflecting limited precision within individual strata. There was no evidence that the association differed according to age, sex, left ventricular ejection fraction category, diabetes mellitus, chronic kidney disease, N-terminal pro-B-type natriuretic peptide category, or atrial fibrillation (all P for interaction ≥0.742). Post-index intensive care unit admission was not examined as a subgroup because it was classified as a secondary outcome (Figure 1; Supplementary Table S4).

**Figure 1:**
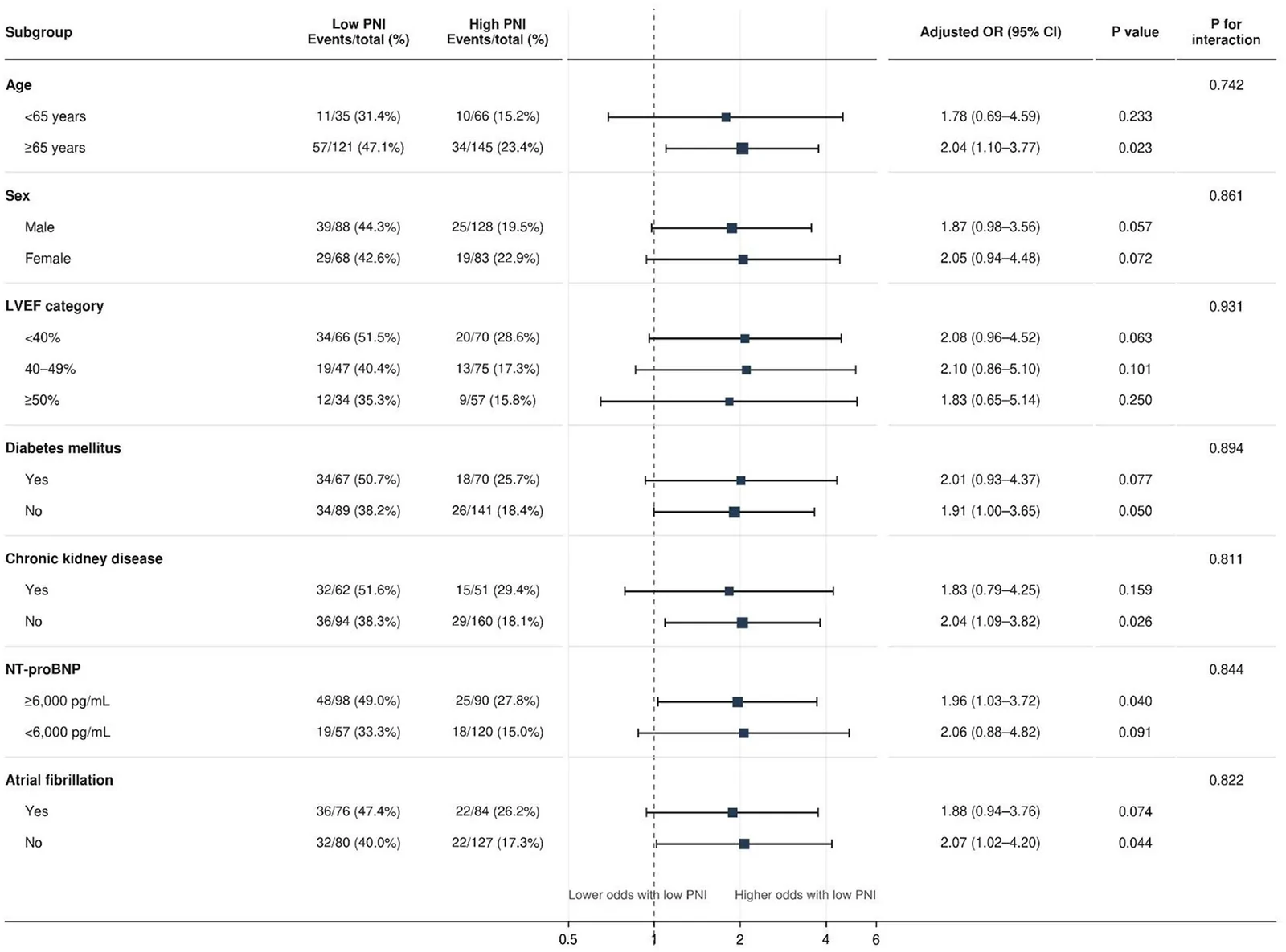
Subgroup analyses of the association between low PNI and the primary composite outcome.

### 3.10 Sensitivity analyses

The association between low PNI and the primary outcome was similar after excluding patients with a component event already present at the index assessment (adjusted OR = 1.88, 95% CI: 1.05–3.36, *P* = 0.033) and in the complete-case analysis (adjusted OR = 1.85, 95% CI: 1.03–3.32, *P* = 0.039). Low PNI was also associated with the hard clinical composite that excluded worsening AHF and resource-utilization events (adjusted OR = 2.06, 95% CI: 1.08–3.91, *P* = 0.028). After patients with C-reactive protein ≥50 mg/L were excluded, the point estimate remained in the same direction but was less precise and the confidence interval included the null (adjusted OR = 1.79, 95% CI: 0.98–3.26, *P* = 0.058) (Supplementary Table S5). A graded pattern was observed across PNI quartiles. Compared with the highest quartile, the lowest quartile was associated with higher adjusted odds of the primary outcome (adjusted OR = 2.57, 95% CI: 1.25–5.29, *P* = 0.010). Each increase of one PNI quartile was associated with lower odds of the primary outcome (adjusted OR = 0.72, 95% CI: 0.58–0.90, *P* for trend = 0.004) (Supplementary Table S6).

### 3.11 Missing data and multiple imputation

PNI, its component variables, and the primary and secondary outcomes were complete for all 367 patients. Missingness was limited for most variables used in the adjusted models, including body mass index (2.5%), systolic blood pressure and heart rate (0.5% each), serum sodium (1.1%), N-terminal pro-B-type natriuretic peptide (0.5%), left ventricular ejection fraction (4.9%), and advanced support status at the index assessment (3.3%). Higher missingness was observed for descriptive right ventricular measurements, including tricuspid annular plane systolic excursion and right ventricular dysfunction (15.5%) and estimated pulmonary artery systolic pressure (17.4%); these variables were not included in the principal multivariable models. Missing covariates in the principal models were handled using multiple imputation by chained equations with 20 imputed datasets. Regression estimates were pooled using Rubin's rules, and a complete-case analysis was conducted as a sensitivity analysis. For subgroup analyses defined by left ventricular ejection fraction or N-terminal pro-B-type natriuretic peptide, subgroup membership was based on observed values, and patients missing the subgroup-defining variable were excluded from the corresponding analysis (Supplementary Table S7).

## 4 Discussion

The present study identified a clinically relevant association between an early, routinely available PNI and incident in-hospital adverse cardiovascular events among patients with AHF presenting to the emergency department. Low PNI remained associated with higher odds of the primary composite outcome after adjustment for demographic characteristics, comorbidities, hemodynamic status, cardiac function, natriuretic peptide concentration, serum sodium, and advanced support at the index assessment. The principal novelty of this analysis lies in its explicit temporal framework, in which PNI was established before outcome ascertainment; its evaluation of a clinically defined in-hospital cardiovascular composite; and its assessment of incremental information beyond conventional variables using discrimination, reclassification, calibration, and bootstrap correction. This extends previous PNI research, which has focused predominantly on mortality during longer-term follow-up, to the immediate emergency and inpatient phase of AHF care (20). Because PNI can be calculated from serum albumin and lymphocyte count without additional testing, it could be incorporated into routine electronic records as an adjunct to early clinical assessment (21, 22). A low value may help identify patients who warrant closer surveillance and formal nutritional evaluation. However, PNI should be interpreted as a complementary prognostic marker rather than a stand-alone triage criterion or direct therapeutic target, because the present retrospective findings establish association and prognostic information rather than the effectiveness of PNI-guided intervention.

The between-group profile suggests that PNI reflects multidimensional physiological vulnerability rather than isolated nutritional deficiency. Patients with low PNI were older, had a lower body mass index, and more often had chronic kidney disease and previous heart failure hospitalization. Their index presentation was also characterized by lower systolic blood pressure and oxygen saturation, higher heart and respiratory rates, more advanced Killip and New York Heart Association classes, and more frequent hypotension, hypoperfusion, and advanced support. Patients with lower PNI showed a laboratory and imaging profile characterized by higher C-reactive protein, creatinine, high-sensitivity cardiac troponin I, and N-terminal pro-B-type natriuretic peptide concentrations, lower estimated glomerular filtration rate and serum sodium levels, reduced left ventricular ejection fraction, and more frequent right ventricular dysfunction and pulmonary congestion, suggesting a greater inflammatory, cardiorenal, and hemodynamic burden. Serum albumin is a negative acute-phase reactant and may vary with nutritional reserve, hepatic synthesis, renal or gastrointestinal loss, capillary permeability, plasma-volume expansion, and systemic or venous congestion (23, 24). Accordingly, a low albumin concentration during AHF should not be interpreted as evidence of inadequate nutritional intake alone. Lymphopenia may reflect neurohormonal stress, endogenous corticosteroid activity, altered lymphocyte trafficking, and immune dysregulation (25). Combining these two components may allow PNI to summarize complementary aspects of host reserve and acute illness severity that are not represented by either measurement in isolation (26). The continuous and quartile analyses further showed a graded pattern across the PNI distribution, arguing against an interpretation based solely on dichotomization at 45. Moreover, the direction of the estimate was retained after excluding patients with C-reactive protein concentrations ≥50 mg/L, although its confidence interval included the null. This finding suggests partial overlap between PNI and marked systemic inflammation but does not support treating PNI as a surrogate for C-reactive protein. Similar findings obtained using the hard clinical composite also indicate that the observed pattern was not confined to treatment-escalation events.

The predictive-performance analyses clarify the amount and type of information contributed by PNI. As a single marker, PNI yielded an AUC of 0.714, with a sensitivity of 60.7% and specificity of 65.5% at the cohort-derived cutoff of 44.8. This represents moderate discrimination and is insufficient for accurate patient classification when used alone (27). The close numerical agreement between 44.8 and the literature-derived threshold of 45 supports the internal coherence of the group comparison; however, the former was selected by the Youden index in the present cohort and should remain distinct from the predefined exposure threshold. Adding continuous PNI to the conventional clinical model increased the AUC from 0.776 to 0.829, corresponding to an increment of 0.053 (95% confidence interval, 0.021–0.085). The accompanying category-free net reclassification improvement of 0.287 and integrated discrimination improvement of 0.061 suggested more favorable directional reclassification and greater separation of predicted probabilities between patients with and without events. Because the AUC can be relatively insensitive to improvement when established predictors are already present, these reclassification measures provide complementary information; nevertheless, none of these indices directly demonstrates improvement in clinical decision-making (28, 29). The combined model also had a lower bootstrap-corrected Brier score than the conventional model (0.151 vs. 0.174), while the corrected calibration slope changed only modestly (0.88 vs. 0.86). Together with the corresponding bootstrap-corrected AUCs of 0.803 and 0.752, these findings indicate a modest but coherent improvement across discrimination and overall prediction error rather than an isolated change in apparent AUC. Finally, adjusted odds ratios across the seven baseline-defined subgroup variables ranged from 1.78 to 2.10, and all interaction *P*-values were at least 0.742. Thus, no heterogeneity in the association was detected according to age, sex, left ventricular ejection fraction, diabetes, chronic kidney disease, N-terminal pro-B-type natriuretic peptide level, or atrial fibrillation. Because several subgroup-specific confidence intervals included 1.00, these results indicate no detected association modification rather than equivalence across clinical phenotypes (30, 31).

Recent evidence generally supports the prognostic relevance of PNI in heart failure, although its performance varies across populations and outcomes. In 805 patients with acute decompensated heart failure, Gu et al. reported an area under the receiver operating characteristic curve (AUC) of 0.715 for PNI alone, closely matching the AUC of 0.714 observed in our cohort; adding PNI and serum chloride increased their conventional-model AUC from 0.807 to 0.857. However, that study excluded in-hospital deaths and focused primarily on longer-term all-cause mortality, whereas our analysis evaluated a strictly post-index composite during hospitalization (14). Conversely, Akça et al. (32) reported limited discrimination for short-term mortality in 104 older patients with acute heart failure (AUC, 0.58), suggesting that the prognostic performance of PNI may depend on age distribution, illness severity, outcome definition, and analytical threshold. El-Sheikh et al. (33) further showed that the association between obesity and 1-year mortality differed according to PNI-defined nutritional status among emergency patients with acute heart failure. Together with the persistence of the PNI association after adjustment for body mass index in our study, this finding suggests that PNI may capture physiological vulnerability not adequately represented by body size alone. Secondary analyses of the Prospective Comparison of ARNI with ACEI to Determine Impact on Global Mortality and Morbidity in Heart Failure Trial (PARADIGM-HF) and the Prospective Comparison of ARNI with ARB Global Outcomes in Heart Failure With Preserved Ejection Fraction Trial (PARAGON-HF) populations similarly demonstrated that lower PNI was associated with greater congestion, frailty, comorbidity burden, and higher rates of cardiovascular death or heart-failure hospitalization across the ejection-fraction spectrum, consistent with our finding of no statistically detectable interaction across left ventricular ejection fraction categories (34). More broadly, a recent meta-analysis of 14 cohorts comprising 14,166 patients found that higher PNI was associated with lower risks of all-cause mortality, cardiovascular mortality, and first heart-failure hospitalization (17). Differences in PNI thresholds, clinical settings, and outcome periods limit direct comparison among studies. Our findings extend this evidence by specifically evaluating incident in-hospital events after early PNI assessment and by demonstrating modest incremental prognostic information beyond conventional variables. Nevertheless, these observational associations do not establish PNI as a causal determinant or therapeutic target.

Several limitations merit consideration. First, the single-center retrospective design remains susceptible to selection bias, residual confounding, and inaccuracies in medical records, although standardized abstraction, independent verification, and blinded outcome adjudication were used. Defining the index PNI time ensured that outcomes followed exposure assessment but cannot establish causality or exclude PNI as a marker of underlying disease severity. Second, PNI was based on a single measurement obtained early after admission, and serial changes were unavailable. PNI also cannot be equated with malnutrition because albumin and lymphocyte count are influenced by inflammation, congestion, fluid status, organ dysfunction, and physiological stress; formal nutritional and frailty assessments were unavailable. Third, some covariates were missing, and multiple imputation relies on the unverifiable missing-at-random assumption. Fourth, the composite included events of differing clinical severity. Worsening AHF requiring treatment escalation may reflect clinician or institutional practice, although the hard-composite sensitivity analysis yielded consistent findings. Fifth, subgroup analyses were exploratory, and several estimates were imprecise. Post-index intensive care unit admission was not examined as a subgroup because it was predefined as a secondary outcome. Sixth, long-term mortality, rehospitalization, functional recovery, and quality of life were not assessed. Finally, the cohort-derived PNI cutoff was exploratory, and relevant thresholds may vary across populations and settings. Although adding PNI modestly improved model performance, this finding does not itself establish clinical utility, and bootstrap validation cannot substitute for external validation. Future multicenter prospective studies should externally validate the predefined PNI threshold and combined model across clinical settings and heart failure phenotypes, including their discrimination, calibration, and clinical net benefit. Serial PNI measurements combined with standardized assessments of nutrition, inflammation, congestion, and frailty may clarify the complementary prognostic information provided by PNI. Longer follow-up and clinical impact studies are also needed before PNI-guided management can be recommended.

## 5 Conclusion

In this retrospective cohort of emergency patients with acute heart failure, low PNI was associated with higher adjusted odds of incident in-hospital adverse cardiovascular events. PNI alone showed moderate discrimination, and its addition to conventional clinical variables provided modest incremental prognostic information in internally validated analyses. PNI may therefore serve as a readily available complementary marker for early risk stratification, but not as a stand-alone decision tool. Prospective multicenter studies with external validation are needed to confirm these findings and establish their clinical utility.

## Data Availability

The raw data supporting the conclusions of this article will be made available by the authors, without undue reservation.
