# Supplementary material for: Association of the prognostic nutritional index with in-hospital adverse cardiovascular events among emergency department patients with acute heart failure
Source: Front Nutr. 2026 Oct 20;13:1949478. doi: 10.3389/fnut.2026.1949478 (PMC13727417; doi:10.3389/fnut.2026.1949478)
Supplement: Supplementary file 1 [file Supplementary_file_1.docx]

Supplementary Table S1. Operational definitions of outcomes

| Outcome | Role in analysis | Operational definition | Assessment window | Data source | Adjudication/overlap rule |
| --- | --- | --- | --- | --- | --- |
| Primary composite endpoint | Primary outcome | First occurrence of all-cause death, cardiogenic shock, sustained VT/VF, cardiac arrest/CPR, acute myocardial infarction, or worsening AHF requiring treatment escalation. | Strictly after index PNI time to discharge/death | Integrated electronic record | Count once per patient; component events may overlap. |
| Hard clinical composite | Sensitivity outcome | First occurrence of all-cause death, cardiogenic shock, sustained VT/VF, cardiac arrest/CPR, or acute myocardial infarction; worsening AHF and resource-utilization outcomes are excluded. | Strictly after index PNI time to discharge/death | Integrated electronic record | Count once per patient; constituent events may overlap. |
| All-cause death | Primary component; secondary outcome | Death from any cause documented during the hospitalization. | Strictly after index PNI time to discharge | Death record and discharge summary | Count once; cardiovascular death is a subset. |
| Cardiovascular death | Secondary outcome | Death due to AHF/cardiogenic shock, acute myocardial infarction, fatal ventricular arrhythmia, sudden cardiac death, or another documented cardiovascular cause. | Strictly after index PNI time to discharge | Death note, discharge summary, ECG/imaging/laboratory record | Two-clinician cause adjudication; not an additional primary component. |
| Cardiogenic shock | Primary component | Persistent SBP <90 mmHg for ≥30 min or vasopressor requirement to maintain SBP ≥90 mmHg, plus clinical or biochemical hypoperfusion attributable primarily to cardiac dysfunction after adequate preload assessment. | New onset strictly after index PNI time | Vital signs, lactate/urine output, medication administration, physician note | Baseline shock is a covariate, not an incident outcome, unless resolved and later recurs. |
| Sustained VT/VF | Primary component | VT lasting ≥30 s or requiring earlier termination because of hemodynamic compromise, or any documented VF. | New onset strictly after index PNI time | 12-lead ECG, telemetry, defibrillator record | Count first episode; exclude isolated nonsustained VT. |
| Acute myocardial infarction | Primary component | Rise/fall in cardiac troponin with ≥1 value above the assay-specific 99th-percentile URL plus clinical evidence of acute myocardial ischemia (symptoms, new ischemic ECG changes/Q waves, new ischemic imaging abnormality, or coronary thrombus). | New diagnosis strictly after index PNI time | Serial troponin, ECG, imaging, angiography, clinician diagnosis | Acute myocardial injury without ischemic evidence is excluded. |
| Worsening AHF | Primary component | New or worsening congestion/hypoperfusion after index requiring escalation beyond the regimen present at index: intensified IV diuretics, addition of a second diuretic, new IV vasodilator/inotrope/vasopressor, new ventilatory support, mechanical circulatory support, or ICU transfer. | Strictly after index PNI time | Medication, respiratory-therapy, procedure, and bed-transfer records | Ongoing unchanged treatment is not an event; overlapping shock/ventilation/ICU events may also be counted as components. |
| Cardiac arrest/CPR | Primary component | Loss of effective circulation requiring chest compressions and/or defibrillation, including pulseless VT/VF, pulseless electrical activity, or asystole. | New onset strictly after index PNI time | Code record, CPR record, telemetry | Count first arrest only; may overlap with VT/VF or death. |
| ICU admission | Secondary outcome | New transfer to an ICU or equivalent critical-care unit after index PNI assessment. | Strictly after index PNI time | Bed-transfer log | Patients already in ICU at index are not counted unless discharged from ICU and later readmitted. |
| Mechanical ventilation | Secondary outcome | New initiation of noninvasive positive-pressure ventilation or invasive mechanical ventilation after index for cardiopulmonary deterioration. | Strictly after index PNI time | Respiratory-therapy and procedure logs | Support ongoing at index is excluded; report invasive ventilation separately if available. |
| Vasoactive support | Secondary outcome | New continuous IV vasopressor or inotrope infusion after index (e.g., norepinephrine, epinephrine, dopamine, dobutamine, milrinone). | Strictly after index PNI time | Medication administration record | Ongoing infusion at index is excluded; isolated IV vasodilator use is not classified as vasoactive support here. |
| Length of stay | Secondary outcome | Elapsed time from hospital admission to discharge or in-hospital death, calculated as total elapsed hours divided by 24. | Admission to discharge/death | Admission-discharge timestamps | Retain fractional days without rounding for analysis; report median (interquartile range). |
| Discharge status | Secondary outcome | Discharged home alive, discharged to rehabilitation/nursing care, transferred for continued care, or in-hospital death. | At hospital exit | Discharge disposition | Mutually exclusive categories; transfer with unknown later outcome is not death. |

Abbreviations: AHF, acute heart failure; CPR, cardiopulmonary resuscitation; ECG, electrocardiogram; ICU, intensive care unit; IV, intravenous; PNI, prognostic nutritional index; SBP, systolic blood pressure; URL, upper reference limit; VT/VF, ventricular tachycardia/ventricular fibrillation.

Supplementary Table S2. Incremental predictive value

| Model comparison | Patients/events | ΔAUC | 95% CI | P value | NRI | 95% CI | P value | IDI | 95% CI | P value |
| --- | --- | --- | --- | --- | --- | --- | --- | --- | --- | --- |
| Combined model vs conventional clinical model | 367/112 | 0.053 | 0.021–0.085 | 0.001 | 0.287 | 0.126–0.448 | <0.001 | 0.061 | 0.034–0.088 | <0.001 |
| Combined model vs PNI alone | 367/112 | 0.115 | 0.074–0.156 | <0.001 | 0.412 | 0.249–0.575 | <0.001 | 0.094 | 0.061–0.127 | <0.001 |

Abbreviations: AUC, area under the receiver operating characteristic curve; CI, confidence interval; IDI, integrated discrimination improvement; NRI, net reclassification improvement; PNI, prognostic nutritional index.

Supplementary Table S3, Panel A. Model specifications

| Model | Purpose | PNI coding | Exact covariates | Patients/events | Missing-data method |
| --- | --- | --- | --- | --- | --- |
| Model 1 | Crude association | Binary (<45 vs ≥45) or continuous per 1 point | PNI only | 367/112 | No missing exposure or outcome |
| Model 2 | Demographic adjustment | Binary or continuous | Age, sex, body mass index | 367/112 | Multiple imputation for body mass index |
| Model 3 | Demographic + comorbidity adjustment | Binary or continuous | Model 2 + diabetes mellitus + chronic kidney disease + previous HF hospitalization | 367/112 | Multiple imputation |
| Model 4 | Fully adjusted association | Binary or continuous | Model 3 + systolic blood pressure + heart rate + LVEF + ln(NT-proBNP) + serum sodium + any advanced support ongoing at index PNI | 367/112 | 20 multiply imputed datasets; pooled with Rubin's rules |
| Conventional prediction model | Baseline risk prediction | Not included | Age + sex + systolic blood pressure + heart rate + peripheral oxygen saturation + LVEF + ln(NT-proBNP) + eGFR + serum sodium + advanced support ongoing at index PNI | 367/112 | 20 multiply imputed datasets |
| Conventional model + PNI | Incremental prediction | Continuous per 1 point | All conventional-model predictors + PNI | 367/112 | 20 multiply imputed datasets |

Abbreviations: eGFR, estimated glomerular filtration rate; HF, heart failure; ICU, intensive care unit; LVEF, left ventricular ejection fraction; NT-proBNP, N-terminal pro-B-type natriuretic peptide; PNI, prognostic nutritional index.

Supplementary Table S3, Panel B. Internal validation

| Model | Apparent AUC | Bootstrap-corrected AUC | Corrected calibration intercept | Corrected calibration slope | Corrected Brier score | Requested resamples | Successful resamples | Failed resamples |
| --- | --- | --- | --- | --- | --- | --- | --- | --- |
| Conventional clinical model | 0.776 | 0.752 | −0.04 | 0.86 | 0.174 | 1,000 | 1,000 | 0 |
| Conventional clinical model + PNI | 0.829 | 0.803 | −0.03 | 0.88 | 0.151 | 1,000 | 1,000 | 0 |

Abbreviations: AUC, area under the receiver operating characteristic curve; PNI, prognostic nutritional index.
Supplementary Table S4. Subgroup analyses for the primary outcome

| Subgroup | Category | Low-PNI events/total | High-PNI events/total | Adjusted OR | 95% CI | P value | P for interaction |
| --- | --- | --- | --- | --- | --- | --- | --- |
| Age | <65 years | 11/35 (31.4) | 10/66 (15.2) | 1.78 | 0.69–4.59 | 0.233 | 0.742 |
| Age | ≥65 years | 57/121 (47.1) | 34/145 (23.4) | 2.04 | 1.10–3.77 | 0.023 | — |
| Sex | Male | 39/88 (44.3) | 25/128 (19.5) | 1.87 | 0.98–3.56 | 0.057 | 0.861 |
| Sex | Female | 29/68 (42.6) | 19/83 (22.9) | 2.05 | 0.94–4.48 | 0.072 | — |
| LVEF category | <40% | 34/66 (51.5) | 20/70 (28.6) | 2.08 | 0.96–4.52 | 0.063 | 0.931 |
| LVEF category | 40–49% | 19/47 (40.4) | 13/75 (17.3) | 2.10 | 0.86–5.10 | 0.101 | — |
| LVEF category | ≥50% | 12/34 (35.3) | 9/57 (15.8) | 1.83 | 0.65–5.14 | 0.250 | — |
| Diabetes mellitus | Yes | 34/67 (50.7) | 18/70 (25.7) | 2.01 | 0.93–4.37 | 0.077 | 0.894 |
| Diabetes mellitus | No | 34/89 (38.2) | 26/141 (18.4) | 1.91 | 1.00–3.65 | 0.050 | — |
| Chronic kidney disease | Yes | 32/62 (51.6) | 15/51 (29.4) | 1.83 | 0.79–4.25 | 0.159 | 0.811 |
| Chronic kidney disease | No | 36/94 (38.3) | 29/160 (18.1) | 2.04 | 1.09–3.82 | 0.026 | — |
| NT-proBNP | ≥6,000 pg/mL | 48/98 (49.0) | 25/90 (27.8) | 1.96 | 1.03–3.72 | 0.040 | 0.844 |
| NT-proBNP | <6,000 pg/mL | 19/57 (33.3) | 18/120 (15.0) | 2.06 | 0.88–4.82 | 0.091 | — |
| Atrial fibrillation | Yes | 36/76 (47.4) | 22/84 (26.2) | 1.88 | 0.94–3.76 | 0.074 | 0.822 |
| Atrial fibrillation | No | 32/80 (40.0) | 22/127 (17.3) | 2.07 | 1.02–4.20 | 0.044 | — |

Abbreviations: CI, confidence interval; LVEF, left ventricular ejection fraction; NT-proBNP, N-terminal pro-B-type natriuretic peptide; OR, odds ratio; PNI, prognostic nutritional index.

Supplementary Table S5. Sensitivity analyses

| Sensitivity analysis | Patients | Events, n (%) | Exposure/comparison | Adjusted OR | 95% CI | P value |
| --- | --- | --- | --- | --- | --- | --- |
| Primary multiple-imputation analysis | 367 | 112 (30.5) | Low vs high PNI | 1.92 | 1.10–3.35 | 0.022 |
| Risk-set analysis excluding patients with any component event present at index PNI measurement | 351 | 101 (28.8) | Low vs high PNI | 1.88 | 1.05–3.36 | 0.033 |
| Complete-case analysis for all Model 4 variables | 334 | 104 (31.1) | Low vs high PNI | 1.85 | 1.03–3.32 | 0.039 |
| Hard clinical composite excluding ICU/resource-utilization events | 367 | 72 (19.6) | Low vs high PNI | 2.06 | 1.08–3.91 | 0.028 |
| Continuous PNI analysis | 367 | 112 (30.5) | Per 5-point increase | 0.70 | 0.56–0.90 | 0.003 |
| Excluding patients with CRP ≥50 mg/L among those with CRP measured | 313 | 91 (29.1) | Low vs high PNI | 1.79 | 0.98–3.26 | 0.058 |

Abbreviations: AHF, acute heart failure; CI, confidence interval; CPR, cardiopulmonary resuscitation; CRP, C-reactive protein; OR, odds ratio; PNI, prognostic nutritional index; VF, ventricular fibrillation; VT, ventricular tachycardia.

Supplementary Table S6. PNI quartile analysis

| PNI quartile | PNI range | Patients | Events, n (%) | Adjusted OR | 95% CI | Wald χ² | P value |
| --- | --- | --- | --- | --- | --- | --- | --- |
| Q4 (reference) | ≥50.2 | 91 | 16 (17.6) | 1.00 | Reference | — | — |
| Q3 | 46.2 to <50.2 | 92 | 25 (27.2) | 1.46 | 0.69–3.09 | 0.983 | 0.322 |
| Q2 | 40.2 to <46.2 | 92 | 32 (34.8) | 1.91 | 0.92–3.96 | 3.032 | 0.082 |
| Q1 | <40.2 | 92 | 39 (42.4) | 2.57 | 1.25–5.29 | 6.615 | 0.010 |
| P for trend | Quartile ordinal score 1–4 | 367 | 112 (30.5) | 0.72 per higher quartile | 0.58–0.90 | 8.210 | 0.004 |

Abbreviations: CI, confidence interval; OR, odds ratio; PNI, prognostic nutritional index.

Supplementary Table S7. Missing-data patterns

| Variable | Available, n | Low-PNI missing, n (%) | High-PNI missing, n (%) | Total missing, n (%) | Analysis role | Handling method |
| --- | --- | --- | --- | --- | --- | --- |
| PNI | 367 | 0 (0.0) | 0 (0.0) | 0 (0.0) | Exposure/grouping | Required for inclusion |
| Albumin | 367 | 0 (0.0) | 0 (0.0) | 0 (0.0) | PNI component | Required for inclusion |
| Lymphocyte count | 367 | 0 (0.0) | 0 (0.0) | 0 (0.0) | PNI component | Required for inclusion |
| Primary composite endpoint/event time | 367 | 0 (0.0) | 0 (0.0) | 0 (0.0) | Primary outcome | Timestamp adjudication |
| All-cause death/event time | 367 | 0 (0.0) | 0 (0.0) | 0 (0.0) | Primary component | Death-record verification |
| Cardiovascular death/event time | 367 | 0 (0.0) | 0 (0.0) | 0 (0.0) | Secondary outcome | Cause adjudication |
| Cardiogenic shock/event time | 367 | 0 (0.0) | 0 (0.0) | 0 (0.0) | Primary component | Clinical/timestamp adjudication |
| Sustained VT/VF/event time | 367 | 0 (0.0) | 0 (0.0) | 0 (0.0) | Primary component | ECG/telemetry adjudication |
| Cardiac arrest/CPR/event time | 367 | 0 (0.0) | 0 (0.0) | 0 (0.0) | Primary component | Code-record adjudication |
| Acute myocardial infarction/event time | 367 | 0 (0.0) | 0 (0.0) | 0 (0.0) | Primary component | Troponin/ischemia adjudication |
| Worsening AHF/event time | 367 | 0 (0.0) | 0 (0.0) | 0 (0.0) | Primary component | Treatment-escalation adjudication |
| Hard clinical composite/event time | 367 | 0 (0.0) | 0 (0.0) | 0 (0.0) | Sensitivity outcome | Derive after component adjudication |
| Post-index ICU admission | 367 | 0 (0.0) | 0 (0.0) | 0 (0.0) | Secondary outcome | Bed-transfer timestamp |
| Post-index noninvasive ventilation | 367 | 0 (0.0) | 0 (0.0) | 0 (0.0) | Secondary outcome | Respiratory-therapy timestamp |
| Post-index invasive ventilation | 367 | 0 (0.0) | 0 (0.0) | 0 (0.0) | Secondary outcome | Procedure timestamp |
| Post-index any mechanical ventilation | 367 | 0 (0.0) | 0 (0.0) | 0 (0.0) | Secondary outcome | Union of NIV and IMV |
| Post-index vasoactive support | 367 | 0 (0.0) | 0 (0.0) | 0 (0.0) | Secondary outcome | Medication timestamp |
| Length of stay | 367 | 0 (0.0) | 0 (0.0) | 0 (0.0) | Secondary outcome | Elapsed hours/24; no rounding |
| Discharge status | 367 | 0 (0.0) | 0 (0.0) | 0 (0.0) | Secondary outcome | Disposition verification |
| Age | 367 | 0 (0.0) | 0 (0.0) | 0 (0.0) | Model 2–4; prediction; subgroup | Complete |
| Sex | 367 | 0 (0.0) | 0 (0.0) | 0 (0.0) | Model 2–4; prediction; subgroup | Complete |
| Body mass index | 358 | 4 (2.6) | 5 (2.4) | 9 (2.5) | Models 2–4 | Multiple imputation; complete-case sensitivity |
| Current smoking | 360 | 3 (1.9) | 4 (1.9) | 7 (1.9) | Baseline/auxiliary | Available-case description |
| Alcohol use | 356 | 5 (3.2) | 6 (2.8) | 11 (3.0) | Baseline characteristic | Available-case description |
| Hypertension | 367 | 0 (0.0) | 0 (0.0) | 0 (0.0) | Baseline; model candidate | Complete |
| Diabetes | 367 | 0 (0.0) | 0 (0.0) | 0 (0.0) | Models 3–4; subgroup | Complete |
| Coronary artery disease | 367 | 0 (0.0) | 0 (0.0) | 0 (0.0) | Baseline/auxiliary | Complete |
| Atrial fibrillation | 367 | 0 (0.0) | 0 (0.0) | 0 (0.0) | Baseline; subgroup | Complete |
| Chronic kidney disease | 367 | 0 (0.0) | 0 (0.0) | 0 (0.0) | Models 3–4; subgroup | Complete |
| COPD | 367 | 0 (0.0) | 0 (0.0) | 0 (0.0) | Baseline characteristic | Complete |
| Previous HF hospitalization | 367 | 0 (0.0) | 0 (0.0) | 0 (0.0) | Models 3–4 | Complete |
| Pre-admission diuretic | 360 | 3 (1.9) | 4 (1.9) | 7 (1.9) | Baseline medication | Available-case description |
| Pre-admission beta-blocker | 360 | 3 (1.9) | 4 (1.9) | 7 (1.9) | Baseline medication | Available-case description |
| Pre-admission ACE inhibitor | 360 | 3 (1.9) | 4 (1.9) | 7 (1.9) | Baseline medication | Available-case description |
| Pre-admission angiotensin receptor blocker | 360 | 3 (1.9) | 4 (1.9) | 7 (1.9) | Baseline medication | Available-case description |
| Pre-admission ARNI | 360 | 3 (1.9) | 4 (1.9) | 7 (1.9) | Baseline medication | Available-case description |
| Pre-admission mineralocorticoid receptor antagonist | 360 | 3 (1.9) | 4 (1.9) | 7 (1.9) | Baseline medication | Available-case description |
| Pre-admission SGLT2 inhibitor | 360 | 3 (1.9) | 4 (1.9) | 7 (1.9) | Baseline medication | Available-case description |
| Pre-admission antiplatelet therapy | 360 | 3 (1.9) | 4 (1.9) | 7 (1.9) | Baseline medication | Available-case description |
| Pre-admission anticoagulant therapy | 360 | 3 (1.9) | 4 (1.9) | 7 (1.9) | Baseline medication | Available-case description |
| Pre-admission statin | 360 | 3 (1.9) | 4 (1.9) | 7 (1.9) | Baseline medication | Available-case description |
| Systolic blood pressure | 365 | 1 (0.6) | 1 (0.5) | 2 (0.5) | Model 4; prediction | Multiple imputation |
| Diastolic blood pressure | 365 | 1 (0.6) | 1 (0.5) | 2 (0.5) | Baseline/auxiliary | Multiple imputation as auxiliary |
| Heart rate | 365 | 1 (0.6) | 1 (0.5) | 2 (0.5) | Model 4; prediction | Multiple imputation |
| Respiratory rate | 367 | 0 (0.0) | 0 (0.0) | 0 (0.0) | Baseline/auxiliary | Complete |
| SpO₂ | 363 | 2 (1.3) | 2 (0.9) | 4 (1.1) | Prediction model | Multiple imputation |
| Temperature | 367 | 0 (0.0) | 0 (0.0) | 0 (0.0) | Clinical status | Complete |
| Killip class III–IV | 367 | 0 (0.0) | 0 (0.0) | 0 (0.0) | Baseline/auxiliary | Complete |
| NYHA class IV | 367 | 0 (0.0) | 0 (0.0) | 0 (0.0) | Clinical status | Complete |
| Pulmonary rales | 367 | 0 (0.0) | 0 (0.0) | 0 (0.0) | Clinical status | Complete |
| Peripheral edema | 367 | 0 (0.0) | 0 (0.0) | 0 (0.0) | Clinical status | Complete |
| Jugular venous distension | 367 | 0 (0.0) | 0 (0.0) | 0 (0.0) | Clinical status | Complete |
| Hypotension | 367 | 0 (0.0) | 0 (0.0) | 0 (0.0) | Baseline/auxiliary | Complete |
| Hypoperfusion | 367 | 0 (0.0) | 0 (0.0) | 0 (0.0) | Baseline/auxiliary | Complete |
| Advanced support ongoing at index PNI time | 355 | 5 (3.2) | 7 (3.3) | 12 (3.3) | Model 4; prediction | Multiple imputation of index-time ongoing status; complete-case sensitivity |
| White blood cell count | 367 | 0 (0.0) | 0 (0.0) | 0 (0.0) | Laboratory description | Confirm zero missingness |
| Neutrophils | 367 | 0 (0.0) | 0 (0.0) | 0 (0.0) | Laboratory description | Confirm zero missingness |
| Hemoglobin | 367 | 0 (0.0) | 0 (0.0) | 0 (0.0) | Laboratory description | Confirm zero missingness |
| Platelets | 367 | 0 (0.0) | 0 (0.0) | 0 (0.0) | Laboratory description | Confirm zero missingness |
| Total protein | 367 | 0 (0.0) | 0 (0.0) | 0 (0.0) | Laboratory description | Confirm zero missingness |
| C-reactive protein | 339 | 14 (9.0) | 14 (6.6) | 28 (7.6) | Sensitivity analysis | Available-case sensitivity analysis |
| Serum creatinine | 364 | 2 (1.3) | 1 (0.5) | 3 (0.8) | eGFR source; auxiliary | Impute creatinine before passive eGFR calculation |
| eGFR | 364 | 2 (1.3) | 1 (0.5) | 3 (0.8) | Prediction model | Passively derive after creatinine imputation; do not independently impute |
| Serum sodium | 363 | 3 (1.9) | 1 (0.5) | 4 (1.1) | Model 4 | Multiple imputation |
| High-sensitivity cardiac troponin I | 357 | 4 (2.6) | 6 (2.8) | 10 (2.7) | Laboratory description | Available-case description |
| CK-MB mass | 367 | 0 (0.0) | 0 (0.0) | 0 (0.0) | Laboratory description | Confirm assay and zero missingness |
| NT-proBNP | 365 | 1 (0.6) | 1 (0.5) | 2 (0.5) | Model 4; prediction; subgroup | Impute for overall models; observed-value subgroup assignment |
| Alanine aminotransferase | 367 | 0 (0.0) | 0 (0.0) | 0 (0.0) | Laboratory description | Confirm zero missingness |
| Aspartate aminotransferase | 367 | 0 (0.0) | 0 (0.0) | 0 (0.0) | Laboratory description | Confirm zero missingness |
| Total bilirubin | 367 | 0 (0.0) | 0 (0.0) | 0 (0.0) | Laboratory description | Confirm zero missingness |
| Prothrombin time | 367 | 0 (0.0) | 0 (0.0) | 0 (0.0) | Laboratory description | Confirm zero missingness |
| International normalized ratio | 367 | 0 (0.0) | 0 (0.0) | 0 (0.0) | Laboratory description | Confirm zero missingness |
| LVEF | 349 | 9 (5.8) | 9 (4.3) | 18 (4.9) | Model 4; prediction; subgroup | Impute for overall models; observed-value subgroup assignment |
| LVEF <40% | 349 | 9 (5.8) | 9 (4.3) | 18 (4.9) | Observed subgroup variable | Derive from observed LVEF; exclude missing LVEF from subgroup analysis |
| Left atrial diameter | 349 | 9 (5.8) | 9 (4.3) | 18 (4.9) | Imaging description | Available-case description |
| LV end-diastolic diameter | 349 | 9 (5.8) | 9 (4.3) | 18 (4.9) | Imaging description | Available-case description |
| TAPSE | 310 | 27 (17.3) | 30 (14.2) | 57 (15.5) | Imaging description | Available-case description |
| Right ventricular dysfunction | 310 | 27 (17.3) | 30 (14.2) | 57 (15.5) | Imaging description | Available-case description |
| Estimated pulmonary artery systolic pressure | 303 | 31 (19.9) | 33 (15.6) | 64 (17.4) | Imaging description | Available-case description |
| Pulmonary congestion on chest imaging | 362 | 2 (1.3) | 3 (1.4) | 5 (1.4) | Imaging description | Available-case description |
| Pleural effusion on chest imaging | 362 | 2 (1.3) | 3 (1.4) | 5 (1.4) | Imaging description | Available-case description |
| Pulmonary edema on chest imaging | 362 | 2 (1.3) | 3 (1.4) | 5 (1.4) | Imaging description | Available-case description |

Abbreviations: AHF, acute heart failure; CPR, cardiopulmonary resuscitation; eGFR, estimated glomerular filtration rate; ICU, intensive care unit; IMV, invasive mechanical ventilation; LVEF, left ventricular ejection fraction; MICE, multiple imputation by chained equations; NIV, noninvasive ventilation; NT-proBNP, N-terminal pro-B-type natriuretic peptide; PASP, pulmonary artery systolic pressure; PNI, prognostic nutritional index; SpO₂, peripheral oxygen saturation; VT/VF, ventricular tachycardia/ventricular fibrillation.
